# Exploring a collaborative approach to the involvement of patients, carers and the public in the initial education and training of healthcare professionals: A qualitative study of patient experiences

**DOI:** 10.1111/hex.13338

**Published:** 2021-08-08

**Authors:** Lesley Scott, Jessica Hardisty, Hannah Cussons, Kathryn Davison, Helen Driscoll, Suzanne Powell, Andrew Sturrock

**Affiliations:** ^1^ Faculty of Health Sciences and Wellbeing, The Sciences Complex, City Campus University of Sunderland Sunderland UK

**Keywords:** interprofessional education, mental health, patient involvement

## Abstract

**Objective:**

This study aimed to explore patients' experiences of their involvement in the design and delivery of interprofessional education interventions focussing on mental ill‐health for students studying in undergraduate healthcare and healthcare‐related programmes.

**Design:**

A qualitative methodology using a Grounded Theory approach was used to undertake an iterative series of focus groups with members of a university's Patient, Carer and Public Involvement (PCPI) Group who have a history of mental ill‐health and were involved in the development and delivery of educational interventions for students on undergraduate healthcare and healthcare‐related programmes. Their experiences of being involved in teaching and learning activities, collaboration with academic staff and integration into the academic faculty were explored. Constant comparative analysis facilitated the identification and prioritisation of salient themes.

**Results:**

Five salient inter‐related themes emerged from the data: (1) reduced stigma and normalisation of experience of illness; (2) enhanced self‐worth; (3) improved well‐being; (4) community and connection; and (5) enduring benefits.

**Conclusions:**

A supportive university community and a designated academic PCPI co‐ordinator facilitate a supportive environment for patients and carers to develop as educators, contribute to the training of future healthcare professionals and improve their own personal well‐being. Appropriately resourced and well‐supported initiatives to integrate patients, carers and the public into the functions of an academic faculty can result in tangible benefits to individuals and facilitate meaningful and enduring connections between the university and the wider community within which it is situated.

**Patient and Public Involvement:**

Patients have been involved in the design of the teaching and learning initiatives that this study was primarily focused on. Patients were given autonomy in determining how their experiences should be incorporated into teaching and learning experiences.

## 1 INTRODUCTION

The importance of engaging patients, carers and the public in supporting the initial education and training of healthcare professionals is well established.^1^, ^2^, ^3^, ^4^ Regulatory bodies require providers of academic programmes to demonstrate that patients are involved in the delivery of teaching and learning and that curriculums are aligned to patient‐centred healthcare policy.^5^, ^6^, ^7^, ^8^ The benefits of patient involvement in healthcare education to the patients themselves, the students they support and academic staff have also been extensively evaluated and described.^9^, ^10^, ^11^ The literature describes numerous examples of teaching sessions, assessments and full academic modules that have been supported by patients.^12^, ^13^, ^14^, ^15^ What is less prevalent are examples whereby full integration of patients, carers and the public into the core functions of an academic faculty has been achieved.^16^, ^17^, ^18^, ^19^

In addition to involving patients in individual academic programmes, increasing attention has been paid to the wider civic responsibilities of universities. Institutions are now being encouraged to examine ‘with purpose and rigour’ how they should fulfil their duties to provide wider public benefit, enrich communities and enhance the connection between universities and places.^20^ This raises questions about how this can be achieved and how the outcomes are measured.

The University of Sunderland made a commitment to fully integrate Patients, Carers and Public Involvement (PCPI) into all the core functions of the Faculty of Health Sciences and Wellbeing, including student admissions, curriculum development and delivery, assessment of students and their preparation to enter the workforce. The faculty is involved in the undergraduate training of a number of future healthcare professionals including nurses, doctors, pharmacists, occupational therapists, physiotherapists and paramedics. The commitment to full integration of patients has extended to the development of the physical resources of the University, including creating a ‘patient flat’, a private facility that acts as a safe space for patients during their engagement in University activities. There is also a full‐time member of academic staff to support the PCPI members and their integration into the faculty, and financial resources to fund their engagement. Individual PCPI group members receive relevant training, including how to provide feedback on student performance and equality, diversity and inclusion requirements. There are currently 160 PCPIs members; a smaller group of their representatives is part of the faculty's governance structure, feeding into Academic Boards and the Faculty Executive. PCPI participants are patients, carers and members of the public who live with, or support people with, long‐term physical and/or mental health conditions. Members are recruited directly from the local community, through patient support groups and charities, and via a relationship developed between the University and the Community Mental Health Team of a local NHS Mental Health Trust. As the project has developed, individuals have also joined through personal recommendation from existing group members. All PCPIs undergo a formal induction and training process facilitated by the lead academic (L. S.) and other experienced PCPI representatives.

In this paper, we evaluate the involvement of PCPIs in an initiative to enhance the multidisciplinary teaching and learning of the interface between mental and physical health in the faculty. Through evaluation of this project, we were also able to explore the wider integration of the PCPI group into the faculty and how this was experienced by individuals within it.

## 2 METHODS

Members of the PCPI group who had a history of mental ill‐health collaborated with a multidisciplinary team of academic staff in the iterative design, development and delivery of a novel interprofessional (pharmacy, psychology and nursing) education (IPE) project. The initiative consisted of two conference‐style events during the academic years 2016/17, 2017/18, which included a team‐based learning case study, an immersive simulation session, a simulated ward activity and a patient narrative session (patients sharing their lived experiences of mental ill‐health). The aim of the sessions was to enhance students' understanding of mental ill‐health, how interprofessional collaboration can enhance the care offered to patients and how parity of esteem between mental and physical ill‐health can be achieved. The session format was iterative and changed over the 2‐year cycle; however, the content and delivery were co‐constructed via collaboration between a team of interprofessional clinical academics and PCPIs. Over the 2‐year period of the IPE project's design and delivery, the university was also investing in the development of the PCPI group, the support available to them and integrating them into the core activities of the faculty including recruitment and assessment.

We adopted a Grounded Theory approach to guide the data collection and analysis, with an iterative process of analysis and further data collection to develop and refine salient themes.^21^ Focus groups were chosen as a means of data collection to facilitate a deeper understanding of the participants' perspectives, and the dynamic nature can generate diverse views and experiences.^22^ We offered individual interviews to those unable to attend a focus group.

### 2.1 Study population and sample

A sample of members of the PCPI group with a history of mental ill‐health was invited, on a voluntary basis, to collaborate with the IPE project and all of these individuals were asked to participate in a qualitative evaluation. Before their involvement in the project, participants attended an informative briefing session with academic staff (L. S. and J. H). A total of 14 members of the PCPI group consented to participate in the project, and all took part in this evaluation.

### 2.2 Data collection

Focus groups with PCPI members who took part in the IPE group aimed to evaluate their experiences of doing so. The broader experiences of the PCPI's of working in the Faculty of Health Sciences and Wellbeing were also explored including their understanding of their role, interactions with each other, students and academic staff and experiences of the working environment. Everyone who took part in focus groups was provided with a participant information sheet (Document S1). An initial topic guide was developed by (L. S.) and (S. P.) that was refined by the multidisciplinary research team (Document S2), serving as a benchmark for exploration during focus groups. An experienced independent qualitative researcher (S. P.) with no involvement in the educational initiative or PCPI group facilitated all focus groups and performed the initial analysis to reduce risk of bias. Focus groups were audio‐recorded and transcribed verbatim to aid qualitative analysis; focus groups took place at the University of Sunderland. Two individual interviews were conducted by the researcher to capture feedback from two participants whose roles were either as a facilitator or as a role‐player, so different from the narrative storytelling. Participants were able to withdraw from the study up until the point that interviews/focus groups were transcribed and incorporated into the data set; no participants withdrew from this study. University Wellbeing services were available for all participants in case any element of the discussion caused distress.

### 2.3 Data analysis

Qualitative data were analysed using a Grounded Theory approach, whereby data were coded and categorized into emergent themes.^21^ Strauss constant comparative analysis facilitated the exploration of new concepts and enrichment of data in subsequent focus groups.^22^, ^23^ Salient themes were identified and a thematic framework was developed by (S. P.); the interprofessional research team individually and then collectively reviewed and refined themes until definitive concepts and final interpretations were agreed. As focus groups took place over a number of months during the design and delivery of the IPE project, the emergence of salient themes during the process facilitated the redesign of the teaching sessions and further exploration of emergent themes during subsequent data collection.

### 2.4 Ethics

Ethical approval was granted by the University of Sunderland Research Ethics Group (reference 005067).

## 3 RESULTS

A total of 14 PCPIs comprising 10 males and 4 females whose ages ranged between 42 and 70 years participated in this project and its evaluation. Three focus groups and two interviews were held between February and December 2017. Focus groups and interviews took place at the University of Sunderland; up to 90 min was designated for either activity to be conducted.

Five salient inter‐related themes emerged from the data^1^: reduced stigma and normalisation of experience of illness^2^; enhanced self‐worth^3^; improved well‐being^4^; community and connection^5^; and enduring benefits.

### 3.1 Reduced stigma and normalisation of experience of illness

All participants in this study had taken part in an initiative within the faculty to enhance the multidisciplinary teaching and learning of the interface between mental health and physical health. Participants therefore had a significant medical history of mental ill‐health or having made significant behavioural changes as a result of living with a long‐term condition(s). Participants reflected on the stigma that they felt was attached to their condition(s) before their involvement with the university PCPI group.

*I couldn't bear to tell anyone. My GP even wrote on my sicknote that it was other issues*. (PCPI 1)

The majority of those interviewed described the opportunity to talk about their condition to students as being beneficial.

*I've found that talking about the, depression, very therapeutic because it, as you're talking to groups about it, you're analysing things yourself and you're seeing how one thing's followed another and why something's happened and what's the best way to recover and that sort of thing. So, I find it very useful for myself as well as for the students*. (PCPI 9)

Participants felt that the interaction with each other provided them with an opportunity to reconcile themselves to their illness and/or diagnosis, which they had struggled to do in other contexts, for example, previous work environments. The regularity of these interactions, facilitated by being part of the PCPI group, provided the opportunity to feel acceptance from peers and normalized their experiences of mental ill‐health.

*It makes you realise… you don't really actually appreciate it that you are quite embarrassed… But when you talk about it and people's reactions are just ‘yes, of course, that's natural, that's normal’, it's just underlining again and again that it is okay*. (PCPI 1)

The interactions with students also facilitated this normalisation and (it was proposed) helped students to understand the range of patient experiences they would be required to engage with when they enter the clinical workforce.

*The diversity of the people that students here are going to be seeing… Cause they're going to walk in, these guys are going to walk in to a pharmacy, they're going to walk in to A&E and they need that (exposure) to these types of people*. (PCPI 3)

Participants felt strongly that it was there role to help students to gain the confidence and skills to work with people with mental ill health.

*I introduce myself; I tell them why I'm here, I'm here to help. I'm here to be the person that hopefully you will be sat in front of in four years' time. And you're here to practice on me. And it's my job to help you be that better person in four years' time*. (PCPI 3)

However, interactions with students were not always positive; lack of preparedness or engagement was felt to be a barrier to the role of the PCPI in supporting students to adopt a greater patient‐centred approach to their learning.

*The ones that are just sat there and quite clearly they haven't read anything, they haven't read the case notes and you're left there sitting thinking ‘well, I'm wasting my time being here completely’*.

### 3.2 Enhanced self‐worth

In addition to providing the opportunity to understand and accept their condition through the relationships and connections made within the University, participants also described how their feelings of self‐worth had been enhanced through their participation. Largely, this appeared to come from a feeling of their role being valued and contributing positively to the development of students. Participants recognized improved feelings of self‐worth in themselves and in other PCPI group members with whom they worked.

*I've seen it in (another PCPI group member)… Absolutely raves about it. Best thing ever… just gets him out, makes him feel valued. That's what it is. It's the feeling valued, the feeling of giving something back. It's just brilliant. Absolutely brilliant. So, presumably, that's the change people have seen in me, I'm now seeing in (another PCPI group member)*. (PCPI 1)

### 3.3 Improved well‐being

All of the participants reported an increase in their feelings of well‐being. This appeared to stem from being provided with the opportunity to contribute to the functions of the academic department and seeing the positive impact that they can have on a student's development. This replaced the loss of purpose felt when previous employment ended or could not be continued due to ill health.

*Not being able to go out of the house… just kills me. I hate it. I absolutely hate it… It's‐ that's coming from depression and anxiety and all that, for me to be able to come and do this, I thought it was great and I loved it. I was buzzing when I got home… I was tired mentally but I was buzzing as well. I really enjoyed it*. (PCPI 12)

### 3.4 Community and connection

The sense of enhanced self‐worth and well‐being that the participants described was often attributed to the sense of community that was facilitated by the University.

*There's not many places really where that's available to you, is there? Where you can do something but in such a supportive environment… You couldn't go into your job and get that supportive environment or even volunteering*. (PCPI 1)

Significantly, participants felt that this community was developed and sustained by the academic lead for the PCPI programme. The knowledge of each individual patient, their history and their ability to contribute to specific sessions was seen as key to the success of the PCPI programme and the welfare of all participants.

*One of the strengths with (the PCPI Facilitator) is that they know every single PCPI by name. They know their journey, what their strengths are, what their weaknesses are… and they have the ability of selecting who would be most suitable for doing different activity… I'm confident that I would never be put in a position I would find too daunting to be able to help the other people*. (PCPI 3)

Participants described the sense of pride that they felt for being part of the PCPI group and the university.

*I tell people I work with the University*. (PCPI 14)

### 3.5 Enduring benefits

The benefits to the participants of being part of the PCPI group were clear; what was also apparent was that these benefits had longevity. Connections between members of the group, with academic staff and students were sustained and enduring. PCPI participants were also able to witness the students' development and see their progress, which contributed to their sense of well‐being and self‐worth.

*I'm on first name terms with them (students). And to see the development… And the progress, and to feel that okay, you're a very small cog in a very large organisation… But it is very rewarding*. (PCPI 2)

## 4 DISCUSSION

Previous research has proposed that involving patients in the design of undergraduate curriculum can facilitate a more patient‐ and student‐centred approach to mental health education.^24^ This study provides further evidence that involvement in undergraduate healthcare education is beneficial to patients with a history of mental ill‐health. The participants included a mixed group, comprising lived experience of either, or both, long‐term mental and physical health conditions. Where participants had mental health conditions, these were mainly long‐term or multiple episodes.

Belonging to a community of patients and carers who work in collaboration with academic staff to deliver teaching and learning can restore patients' sense of identity, help them to normalize their health problems and reduce the stigma that patients themselves can associate with mental ill‐health.^13^ In our study, the majority of the PCPI participants are paid an hourly rate for their involvement and have access to staff cards, a university email address and library access as part of the benefits of involvement.

The benefits of collaborative working with patients have been described in previous studies, with more recent examples that link both IPE and the potential to reduce mental illness stigma.^2^, ^12^, ^13^ The NHS Long Term Plan^25^ sets out a clear direction for the future NHS and places greater emphasis on service integration, including new models of care combining primary and secondary care and health and social care. The project that has been described here promoted not only collaboration between patients and academic staff but also cross‐faculty cooperation, bringing together those from a range of healthcare professional backgrounds to deliver joint teaching and learning initiatives in a move towards replicating the desired models of integrated healthcare. Since its inception, the project has endured beyond the initial development stage and has now expanded to include other health and social care professionals in training.

Previous studies have outlined the challenges faced during attempts to achieve partnership between patients and academic faculties in the delivery of undergraduate education.^16^ Efforts are often thwarted by difficulties in achieving representativeness and meaningful engagement and problems arising from an ‘inequality of power’ between service users and educationalists. Shortcomings in the supportive arrangements available to patients also hamper full inclusion into academic faculties.^26^ Although the claim cannot be made that all of these issues have been solved by the resources and infrastructure put in place by the University of Sunderland, the results of this study show that some have been mitigated for. A dedicated member of staff to support patients and carers and act as their advocate within the faculty appears to be essential to the success of attempts at integration. This has also been important when constructing a response to the negative experience of patients when interacting with students who are difficult to engage. This has been addressed by the lead academic who has developed training for PCPI group members on how they can encourage participation, and work has also been done across the faculty to better prepare students to work with patients from the early years of their training.

As universities consider how they can meet their responsibilities to the wider communities in which they are situated, we have outlined an attempt by which meaningful engagement of patients can be achieved in a socially inclusive way. Working with undergraduate healthcare students provides a unique opportunity for those who may be facing isolation due to illness to re‐establish enduring connections with others and gain new skills. These benefits have been shared with mental health NHS Trusts and a formal referral mechanism is now in place to facilitate patients who it is felt may benefit from joining the PCPI group to do so.

### 4.1 Limitations

The findings of this study relate directly to patients who have clearly demonstrated a willingness to become involved in educating healthcare students. Therefore, findings may not be generalisable to all patients with mental ill‐health in other settings. The majority of PCPIs are paid for their participation in teaching sessions, which may potentially result in a positive bias towards the university.

### 4.2 Future work

Further exploration of referral pathways and means of identifying patients who may receive the most benefit from involvement in education could further enhance outcomes and patient care. Our study has focused on the therapeutic benefits associated with mental ill‐health or long‐term conditions; further work should look to explore the potential benefits to patients with other health conditions who are involved with undergraduate education.

## 5 CONCLUSION

Appropriately resourced and well‐supported initiatives to fully integrate patients, carers and the public into the functions of an academic faculty can result in tangible benefits to individuals and facilitate meaningful and enduring connections between the university and the wider community within which is it is situated. This study suggests that a supportive community of patients and carers, contributing to interprofessional undergraduate healthcare education, can provide enduring benefits to its members. Universities should provide sufficient resources to support patient involvement in undergraduate education as a means of enriching the student curriculum and empowering patients to improve health outcomes. Clinicians and health charities should consider implementing referral pathways for appropriate patients and further explore collaborative opportunities to improve the health of patient volunteers.

Key to the success of patient involvement in an undergraduate setting is a supportive environment led by a dedicated member of academic staff; the importance of this role and a good understanding of each individual patient's history and ability to contribute to appropriate sessions are key to the welfare of the patient involved and the success of a patient‐led curriculum.

## CONFLICT OF INTERESTS

The authors declare that there are no conflict of interests.

## AUTHOR CONTRIBUTIONS

Jessica Hardisty, Andrew Sturrock, Lesley Scott and Helen Driscoll designed the study. Hannah Cussons performed the literature search. Suzanne Powell performed the focus groups and interpreted the data. Jessica Hardisty, Andrew Sturrock, Lesley Scott, Helen Driscoll, Kathryn Davison and Suzanne Powell reviewed and refined the data. Jessica Hardisty and Andrew Sturrock wrote the paper, and all authors revised it.

## Supporting information

Supporting information.

Supporting information.

## Data Availability

Research data are not shared.
