# Supplementary material for: Exploring a collaborative approach to the involvement of patients, carers and the public in the initial education and training of healthcare professionals: A qualitative study of patient experiences
Source: Health Expect. 2021 Aug 8;24(6):1988–94. doi: 10.1111/hex.13338 (PMC8628596; doi:10.1111/hex.13338)
Supplement: Supplementary file 1 — Supporting information. [file HEX-24-1988-s001.docx]

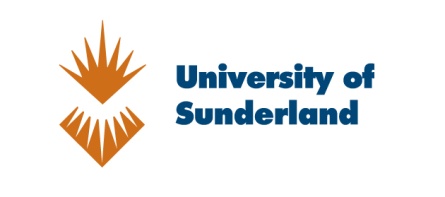


**Interprofessional education conference**

**Information Pack**

Contents

1. Introductory letter, including confirmation of date and time of attendance
2. Information Sheet
3. Consent form


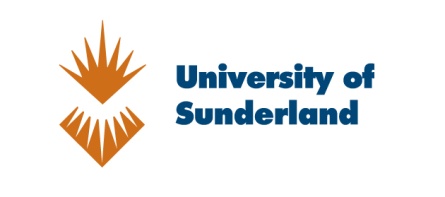


Dear Participant

Thank you for agreeing to attend a feedback session with regard to the interprofessional education conference (Pharmacy, Psychology, Counselling and Public Health) you were part of in December 2017. The findings from these feedback sessions will be used to inform the structure and content of similar future seminars. Your suggestions as to how the experience could be improved or developed further in the future will be gratefully received.

We would like to remind you that taking part in these sessions is completely voluntary and that you can contribute as much or as little to the feedback sessions as you wish.

Your feedback session will be held on [*insert date and time*], in [*venue*], and we look forward to seeing you then. The feedback session is likely to last between 45 minutes and 1 hour. The feedback sessions are being run by an external research associate (Suzanne Powell) with experience of interprofessional education evaluation.

Once you have read through this pack, if you have any questions, please do not hesitate to either email me or give me a quick ring and I will be happy to answer any of your questions. As part of this pack is a consent form, if you are happy with the information set out in this letter and information sheet, then when you attend your feedback session you will be asked to sign a copy which we will provide for you.

Thank you for taking the time to read this information and agreeing to participate

Yours faithfully


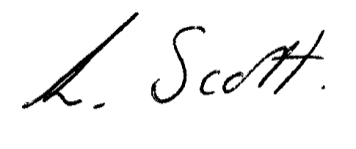


Dr Lesley Scott

Senior Lecturer: Patient, Carer and Public Involvement

Department of Pharmacy, Health and Well-being

Faculty of Applied Sciences

University of Sunderland, The Sciences Complex

Chester Road

Sunderland SR1 3SD

Tel: 01915152388

Email: [l.scott@sunderland.ac.uk](mailto:l.scott@sunderland.ac.uk)


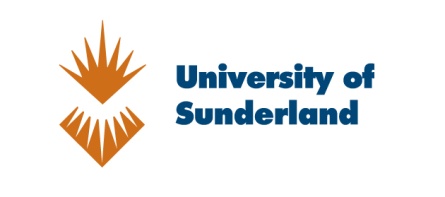


**Interprofessional education (IPE) conference**

**Information Sheet for participants**

**Background to IPE**

At the University (name removed for peer review) we run a number of interprofessional conference style events, some of which include patient, carer and public involvement (PCPI). The interprofessional education conference which involved students from Pharmacy, Psychology and Counselling was focused on IPE collaboration in the preparation and delivery of pre-registration students focusing on patients with mental ill health.

Patients with mental ill health have poorer physical health than the general population. Despite being well known to the healthcare system & having many health encounters they access emergency NHS services significantly more than people without mental ill health, with the majority of the care sought for physical health conditions. It is suggested that opportunities for planned, integrated care & the identification & support of physical health needs are lost. This project aims to address the requirement for well-designed inter-professional teaching & learning opportunities at undergraduate level aimed at equipping trainee healthcare professionals with the knowledge & skills to effectively support patients with mental ill health when they enter the clinical workforce. This project will place an equal focus on the physical & mental health needs of this patient group. Healthcare providers require employees with these skills if the disparity between the mortality & morbidity of these patients and the general population is to be minimised.

**Aims & objectives of the Project**

- To develop inter-professional teaching & learning which prepares pharmacy, nursing & psychology undergraduates for work with patients with mental ill health.
- To replicate the post-qualification focus on multidisciplinary collaboration in the care of patients with mental ill health in undergraduate programmes.
- To involve patients, service users & carers in designing undergraduate mental health education.
- To enhance collaboration between pharmacy, nursing, & psychology academic staff in the delivery of teaching & learning around mental health.
- To engage public & private sector employers & 3rd sector organisations in the delivery of undergraduate teaching & learning.
- To produce teaching & learning materials which enable long term initiative sustainability.

**Invitation**

As a student/PCPI participant who has attended the IPL Mental Health conference, thank you for agreeing to provide feedback and thoughts regarding the conference you were involved with in order to shape future development. Your participation within this evaluation is entirely voluntary and although you have consented to participate, you can withdraw at any time before 1st March 2018 (when data analysis will commence) without any consequence. If you are not comfortable in the feedback session, you can leave at any time without this affecting you.

**Sharing your experience**

Thank you for agreeing to participate in this evaluation. It is anticipated that the feedback session, which will be held with other students/PCPI participants will last for between 45 and 60 minutes. The feedback session will explore your experiences of participating (4^th^ and 8^th^ December 2017) in the IPE mental health conference which involved PCPI participants as well as any suggestions for further development. The feedback session will be audio‐recorded with your permission. The data will be stored securely (name removed for peer review), where only the principal investigator and two co-investigators will be able to gain access to it. All recordings will be anonymised and confidential and your personal details will remain confidential and you will not be identifiable within reports or journal publication of findings.

**Consent and participant rights**

You will be able to change your mind and withdraw your recording at any time before 1^st^ March 2018. You can contact Lesley Scott at the address at the end of this information sheet to do this.

Your name and any personal details will remain completely confidential.

**Contact**

If you have any questions, please contact:

Dr Lesley Scott

Senior Lecturer: Patient, Carer and Public Involvement

Department of Pharmacy, Health and Well-being

Faculty of Applied Sciences

University of Sunderland

The Sciences Complex

Chester Road

Sunderland SR1 3SD

Tel: 01915152388

Email: [l.scott@sunderland.ac.uk](mailto:l.scott@sunderland.ac.uk)

*Thank you for thinking about taking part in this evaluation project and for taking the time to read this information sheet. Please note that this information sheet can be made available in appropriate accessible formats.*


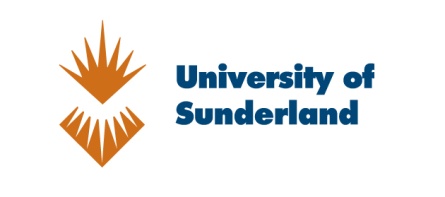


**Consent Form**

**Interprofessional education (IPE) conference: Pharmacy, Psychology, Counselling and Public Health Feedback Sessions**

|  | Please **initial** the boxes below **to confirm** |
| --- | --- |
| I, ………………………………………(***put your name here*)** have read and understood the information sheet on Interprofessional education (IPE) conference: Pharmacy, Psychology, Counselling and Public Health feedback sessions. |  |
| I have had the opportunity to think about the information and ask questions of (name removed for peer review) and I am happy with the answers she gave me. |  |
| I understand that participating in this research is voluntary and that I am free to withdraw my material at any time before 1^st^ March 2018 by contacting (name removed for peer review) at the address below. |  |
| I give permission for (name removed for peer review) to use the information I provide during the feedback session within any report or journal article generated as a result of this feedback. I understand that my personal details will remain confidential and I will not be identifiable within any of the thesis findings. |  |

Name of interviewee [PLEASE PRINT] _______________________________________

Signature………………………………………………………… Date………………….

Name of interviewer ____ _______________________________________

Signature …………………………………………………………. Date……………………

**Contact**

Dr Lesley Scott: Senior Lecturer: Patient, Carer and Public Involvement

Department of Pharmacy, Health and Well-being

Faculty of Applied Sciences

University of Sunderland

The Sciences Complex

Chester Road

Sunderland SR1 3SD

Tel: 01915152388

Email: [l.scott@sunderland.ac.uk](mailto:l.scott@sunderland.ac.uk)
