# Supplementary material for: Exploring a collaborative approach to the involvement of patients, carers and the public in the initial education and training of healthcare professionals: A qualitative study of patient experiences
Source: Health Expect. 2021 Aug 8;24(6):1988–94. doi: 10.1111/hex.13338 (PMC8628596; doi:10.1111/hex.13338)
Supplement: Supplementary file 2 — Supporting information. [file HEX-24-1988-s002.docx]

**Interprofessional education conference topic Guide**

3 sessions to attend (45 mins each) – ward based; immersive simulation; patient session

To explore the interactions with the different student groups – e.g. nursing, psychology, public health and pharmacy – around the focus for their questions.

1. Overall impression of your experience
2. Did you feel you had enough time with each group?
3. Did you feel the students were engaged in the session? Had they prepared for the session? Did they understand its purpose?
4. Were the students engaging with one another, across professional groups?
   - 1. Any examples where this worked well / did not work so well – why?
     2. Could you identify which professional groups the students belonged to? In what way?
5. What sort of questions were being asked of you?
6. Did they focus on a particular element of your treatment?
7. Was there anything that was missed?
8. Were there any questions which were completely irrelevant?
9. Were there any silences? How did you cope with these?
10. How do you feel you were treated by the students? Did you feel valued? Did they behave in an appropriately professional manner?
11. Was the number of students in each group manageable?
12. What was your experience of how the day was scheduled? Pace, physical space, facilities etc
13. Did you receive appropriate support from academic staff? How might this be improved?
14. Do you feel that being involved in this round of interprofessional learning has impacted on you and your condition? If yes, in what ways? Positive and negative.
15. Is there anything I haven’t covered that you’d like to add?
